# Musculoskeletal disorders among waste pickers in sorting cooperatives in São Paulo, Brazil

**DOI:** 10.47626/1679-4435-2024-1352

**Published:** 2025-08-25

**Authors:** Marcia Cristina Castanhari Mandelli, Rafael Junqueira Buralli, Felipe Parra do Nascimento, Nelson Gouveia

**Affiliations:** 1 Department of Preventive Medicine, Faculdade de Medicina, Universidade de São Paulo, São Paulo, SP, Brazil

**Keywords:** waste pickers, recycling, working conditions, cumulative trauma disorders, musculoskeletal pain., catadores, reciclagem, condições de trabalho, transtornos traumáticos cumulativos, dor musculoesquelética.

## Abstract

**Introduction:**

Waste pickers are exposed to numerous occupational risks, including ergonomic risks.

**Objectives:**

To verify the occurrence of self-reported musculoskeletal disorders and associated risk factors among waste pickers working in sorting cooperatives.

**Methods:**

This cross-sectional study included a convenience sample of waste pickers from four cooperatives in metropolitan São Paulo, Brazil. Observations were made of their work environments and processes; a questionnaire was applied to characterize socioeconomic and organizational status, as well as the Nordic Musculoskeletal Questionnaire. After descriptive analysis, univariate and multivariate regression were used to identify risk factors associated with work-related pain and discomfort in the last 12 months.

**Results:**

A total of 250 waste pickers were included, mostly women (62%) aged 41 to 59 years (53%) who were Black or of mixed race (66.4%) and had low education. There was a high prevalence of musculoskeletal disorders among waste pickers from all cooperatives, with 163 (65%) reporting pain and discomfort. The cooperatives had similar production processes, but different structures and operating conditions. Working in a cooperative with worse infrastructure, prior work-related accidents, and having a prior occupation were identified as the main risk factors for musculoskeletal disorders.

**Conclusions:**

Cooperatives with precarious structure and working conditions partially explain the high proportion of waste pickers with musculoskeletal disorders, reinforcing the need for initiatives and policies to promote decent and safe work, thus improving the health and quality of waste pickers.

## Abstract (pt)

**Introdução:**

Catadores de recicláveis estão expostos a diversos riscos ocupacionais, inclusive ergonômicos.

**Objetivos:**

Verificar a ocorrência de distúrbios osteomusculares autorreferidos e os fatores associados entre catadores de materiais recicláveis que atuam em cooperativas de triagem.

**Métodos:**

Estudo transversal, com amostragem por conveniência, com catadores de quatro cooperativas da região metropolitana de São Paulo. Foram realizadas observações dos ambientes e processos de trabalho, além da aplicação de um questionário para caracterização socioeconômica e organizacional e do Questionário Nórdico de Sintomas Osteomusculares. Realizou-se análise descritiva, seguida de regressão univariada e multivariada para a identificação dos fatores associados à dor e ao desconforto relacionados ao trabalho nos últimos 12 meses.

**Resultados:**

Foram incluídos 250 catadores, com predomínio do sexo feminino (62%), idade entre 41-59 anos (53%), raça/cor negra/parda (66,4%) e baixa escolaridade. Observou-se elevada prevalência de distúrbios osteomusculares entre catadores de todas as cooperativas avaliadas, sendo dor e desconforto referidos por 163 catadores (65%). Todas as cooperativas possuíam processos produtivos semelhantes, porém com estrutura e condição de funcionamento diferentes. Trabalhar em uma cooperativa com pior estrutura, ter tido acidente de trabalho e ocupação pregressa foram identificados como os principais fatores de risco para distúrbios osteomusculares.

**Conclusões:**

Cooperativas com estrutura e condições de trabalho precárias explicam, em parte, a elevada proporção de catadores com distúrbios osteomusculares, reforçando a urgência de ações e políticas para promover o trabalho decente e seguro, além de melhorar a saúde, a qualidade de vida e as condições de trabalho dos catadores de materiais recicláveis.

## INTRODUCTION

The increasing generation of urban solid waste has become one of the most pressing environmental issues of our time. It is estimated that São Paulo alone, the largest city in South America, with its more than 12 million inhabitants, produces around 21,000 tons/day of waste, of which 12,000 are household waste.^1,2^ In general, urban waste management infrastructure is still insufficient and continues to be a key issue in the contemporary political scenario, especially in metropolitan regions.^3^

In this context, recyclable material collectors have been present in Brazil since the beginning of the 20th century, initially in the form of bottle collectors and, more recently, as workers involved in solid waste management through cooperatives or collection associations, which were included in the National Solid Waste Policy of 2010.^4^ In 2016, the city of São Paulo had a network of 20 sorting centers in the Solidary Selective Collection Program, totaling approximately 1,000 collectors.^5^ In 2020, 25 waste picker cooperatives were registered in the municipal selective collection program, generating income for the families of around 940 waste pickers.^6^ But it is difficult to accurately estimate the number of active waste pickers, and the number above is likely an underestimation, given the high level of informal employment in the sector.

Although waste picking is an important mechanism for social inclusion through organized work and income generation, as well as for urban cleaning and public health, waste pickers commonly face social exclusion, precarious work processes and difficulty performing their activities.^7^ Considering that only a quarter of Brazilian municipalities have waste separation systems, the high recycling rates in this country, especially of cans and cardboard, are due to the work of waste pickers.^8^

These workers face several occupational risks when collecting recyclable materials on the streets and in cooperatives and associations, such as exposure to physical, chemical, biological, and biomechanical agents, lifting and carrying heavy loads, performing repetitive movements, inadequate posture, and intense physical effort. They also face physical risks, such as work accidents due to cuts, punctures, being run over, falling, animal bites, and using inappropriate tools and machinery.^9,10^

Several factors related to the work environment and work processes can result in biomechanical risk and can affect the health of waste collectors, such as the infrastructure and condition of the cooperatives, the availability and use of equipment, work organization, repetitive movements, excessive work hours, remuneration for productivity, and an accumulation of tasks.^11^

Waste pickers often perform manual work in confined spaces, with inadequate equipment and biomechanical risk factors. As a result, some studies of waste pickers have found that they have a high prevalence of musculoskeletal disorders, mainly referred to as pain or discomfort in the spine and upper and lower limbs.^12-16^

Nevertheless, there have been few studies on recyclable material collectors in Brazilian cooperatives to determine the main risk factors they face and the problems that most commonly compromise their health and quality of life. Therefore, this study aimed to estimate the prevalence of work-related musculoskeletal pain or discomfort among recyclable material collectors in São Paulo, analyzing associated factors that can inform public policy regarding disease prevention and health promotion among these workers.

## METHODS

A cross-sectional study was conducted using non-probabilistic convenience sampling to estimate the frequency of musculoskeletal pain/discomfort and associated factors among recyclable material collectors in metropolitan São Paulo. Initially, 10 cooperatives registered with the *Movimento Nacional dos Catadores de Materiais Recicláveis* (National Recyclable Material Collectors Movement) were visited to observe the general work organization and waste types handled. Of these, based on their availability and location, four were selected for a detailed analysis of work environments and processes, including interviews with the collectors and analysis of work organization, waste types, and affiliation with the São Paulo municipal collection program.

All workers over 18 years of age in the cooperatives were eligible to participate in the study, regardless of sex, race, age, or function (e.g., collection, receiving, sorting, transportation, pressing, storage, administration, cleaning, etc.). No exclusion criteria were established, and all collectors who were working at the four selected cooperatives on the days of data collection (between August and December 2016) were invited to participate. Since no one refused, all workers present were included in the study.

The cooperatives’ work conditions were observed and described, including the production system (semi-mechanized or manual), the availability and capacity of the collection truck, storage container types, the availability and quantity of equipment (conveyors, presses, and wheelbarrows) and the operating conditions of the equipment (operating, partially operating, or offline).

While the participating waste pickers were at their workplace and performing their tasks, semi-structured interviews were conducted regarding sociodemographic characteristics, such as sex, age, race, region of origin, and education; work activities, including work history, previous occupations, characteristics of work organization (posture, physical effort, monotony, repetitiveness, production count, shift rotation, and breaks) and the occurrence of work accidents. The interview was based on a questionnaire about musculoskeletal symptoms in hairdressers that had been adapted for waste pickers. The Nordic Musculoskeletal Questionnaire, validated for use in Brazil,^16^ was also used to determine the presence of at least one episode of musculoskeletal pain/discomfort that could be work-related in the 12 months prior to the interview.

The responses were double-entered and a consistency analysis was performed to identify any typing and coding errors. Descriptive analysis of the variables was followed by logistic regression models, considering work-related musculoskeletal pain/discomfort in the 12 months prior to the study as the dependent variable and factors associated with pain/discomfort as independent variables.

Univariate and multivariate logistic regression models were performed to identify factors associated with the outcome. Independent variables with p ≤ 0.2 in the univariate analysis were considered in the multivariate models (stepwise), with only statistically significant variables (p < 0.05) maintained in the final model. All analyses were performed in Stata 14.

Based on our findings and the available literature, urgent public actions and policies aimed at recyclable material collectors in Brazil are proposed. The protocol for this study was approved by the Faculdade de Medicina da Universidade de São Paulo Ethics Committee (decision 362/2015, August 14, 2015). Participation in the study was voluntary and all individuals provided informed consent.

## RESULTS

A total of 250 waste pickers from four cooperatives in metropolitan São Paulo were interviewed, ranging from 47 to 71 workers in each (median = 66) cooperative. The cooperatives had been operating for an average of 13 years, and the majority (75%) ran full-time. The average length of worker employment in the cooperatives was 6 years, and 84% reported having had another occupation before becoming a waste picker. The most commonly mentioned occupations were general labor and domestic worker.

The majority (62%) of the interviewed waste pickers were women, with only one cooperative having a majority of men. In all cooperatives, the majority (65%) of the waste pickers were > 41 years of age. Overall, the average age was 44 years (range 20 to 74). The majority identified themselves as Black or of mixed race (66%) and had a low education level, with 77% being illiterate or having only completed 8th grade. Similar numbers of workers came from the southeastern/southern and the northeastern/northern/midwestern regions of Brazil (Table 1).

**Table 1:** Sociodemographic characteristics of waste collectors in four recyclable materials cooperatives in metropolitan São Paulo, 2016

Variables	A	B	C	D	Total
Sex					
Female	33 (70)	41 (65)	32 (45)	48 (70)	154 (62)
Male	14 (30)	22 (35)	39 (55)	21 (30)	96 (38)
Age (years)					
19-29	1 (2)	10 (16)	9 (13)	16 (23)	36 (14)
30-40	6 (13)	13 (21)	19 (27)	15 (22)	53 (21)
41-59	33 (70)	35 (55)	32 (45)	34 (49)	134 (54)
≥ 60	7 (15)	5 (8)	11 (15)	4 (6)	27 (11)
Race					
White	18 (38)	21 (33)	23 (32)	22 (32)	84 (34)
Black/mixed	29 (62)	42 (67)	48 (68)	47 (68)	166 (66)
Region of origin					
Southeast or South	23 (49)	35 (56)	36 (51)	35 (51)	129 (52)
Northeast, North, or Midwest	24 (51)	28 (44)	35 (49)	34 (49)	121 (48)
Education					
None	3 (6)	4 (6)	7 (10)	7 (10)	21 (8)
1st-4th grade	22 (47)	22 (35)	20 (28)	23 (33)	87 (35)
5th-8th grade	9 (19)	27 (43)	28 (39)	20 (29)	84 (34)
High school or college	13 (28)	10 (16)	16 (23)	19 (28)	58 (23)
Total	47	63	71	69	250 (100)

Waste pickers usually performed all activities in the production process, e.g., collecting, receiving, sorting, pressing, and storing waste. They were often exposed to various risks related to load (weight and physical effort), pace (rhythm and monotony), movement (repetitive or not), physical risk (falls, accidents, injuries, and shocks), accidents involving animals, such as spider, rat, dog, and cat bites and scorpion stings, in addition to psychosocial risks (precarious and unrecognized work, monotony, income instability, informal employment, and lack of social protection), a precarious work environment, and a lack of collective and individual protective equipment.

The majority of workers (65%) reported episodes of pain or discomfort in the last 12 months, with a prevalence of 83% in cooperative A, approximately 70% in cooperatives B and C and 42% in cooperative D. Regarding the location of the pain, the lower back (50%), upper limbs (45%) and lower limbs (31%) were the most prevalent. Overall, 34% of the workers reported occupational accidents (n = 86), ranging from 45%, 42%, 35% to 19% in cooperatives A, C, B, and D, respectively.

Regarding the cooperatives’ infrastructure and operation parameters, all had semi-mechanized means of production, including a collection truck, a conveyor belt, a press, and wheelbarrows. However, the quantity and operating conditions varied. Most cooperatives (A, B and C) used cage trucks, with only one (D) using a compactor truck (Table 2). The compactor model facilitates dumping a larger quantity of waste at a single time into the conveyor silo, reducing physical effort and the number of movements and repetitions, while the cage model requires workers to manually dump the waste into the silos, bag by bag, increasing the risk of injuries and musculoskeletal disorders. Regarding equipment condition (i.e., the conveyor belt, press, and wheelbarrow), cooperative D had the largest quantity of equipment and all of it was in operation. In cooperative A, all of the equipment was offline, while in cooperatives B and C it was partially operating (Table 2).

**Table 2:** Characteristics of the production process and working conditions in four recyclable materials cooperatives in metropolitan São Paulo, 2016

Characteristics	Cooperatives			
Means of production	A	B	C	D
Cage truck	4	5	6	0
Compactor truck	1	2	1	6
Standardized storage container	No	PO	PO	Yes
Conveyor belt	1	2	2	4
Press	2	2	3	5
Wheelbarrow	1	2	3	5
				
Conditions of use of equipment				
Conveyor belt	1 offline	1 operating 1 PO	2 operating	4 operating
Press	2 offline	1 operating 1 offline	2 operating 1 PO	5 operating
Wheelbarrow	1 offline	1 operating 1 offline	2 operating 1 PO	5 operating

PO = partially operating.

Regarding waste storage, the cooperatives varied between polyethylene bags and standardized containers (wheeled steel containers for large bags and 200-liter metal or plastic drums), which facilitate storage and moving larger quantities of waste. Polyethylene bags expose workers to greater ergonomic risks, physical effort, and repetitive movement.

In all cooperatives, the collectors themselves performed administrative activities, such as determining the number of collectors assigned to each task, recording the quantities of waste collected, sorted, and sold, equipment maintenance, worker recruitment, paying fees, monitoring work absenteeism, and obtaining licenses for collectors. As a result, the workers face psychosocial risks, overload, and pressure, and an accumulation of tasks that can result in muscle pain/discomfort.

It was observed that women, workers aged ≥ 60 years, and Black or mixed-race workers were more likely to report pain/discomfort, although there was no significant difference between comparison groups. There was also no significant association between the region of origin, education level, and reported pain or discomfort (Table 3).

**Table 3:** Association between pain/discomfort in the last 12 months and sociodemographic characteristics among recyclable material collectors from cooperatives in metropolitan São Paulo, 2016

Variables	Pain/discomfort n (%)	OR	95%CI	p-value
Sex				
Female	58 (61)	1.00	Ref.	Ref.
Male	105 (68)	1.37	0.80-2.32	0.249
Age (years)				
19-29	36 (14)	1.00	Ref.	Ref.
30-40	53 (21)	0.97	0.40-2.29	0.753
41-59	134 (54)	0.77	0.36-1.64	
≥ 60	27 (11)	1.13	0.41-3.12	
Race				
White	49 (58)	1.00	Ref.	Ref.
Black/mixed	114 (69)	1.56	0.90-2.69	0.107
Region of origin				
Southeast or South	81 (68)	1.00	Ref.	Ref.
Northeast, North, or Midwest	82 (63)	1.24	0.73-2.10	0.409
Education				
None	12 (57)	1.00	Ref.	Ref.
1st-4th grade	58 (67)	1.50	0.56-3.96	0.612
5th-8th grade	58 (69)	1.67	0.62-4.45	
High school or college	35 (60)	1.14	0.41-3.13	

OR = odds ratio.

The waste pickers from cooperative D were significantly less likely to report musculoskeletal pain or discomfort than those from the other cooperatives. The highest prevalence of musculoskeletal pain or discomfort was found among waste pickers from cooperative A, whose odds were 5.6 times higher than in cooperative D, while the odds were 3 times higher in the other cooperatives. Waste pickers who reported a prior occupation or who suffered a prior occupational accident also had a significantly greater chance of musculoskeletal pain or discomfort (Table 4).

**Table 4:** Association between pain/discomfort in the last 12 months and production processes, work history, and work organization among recyclable material collectors from cooperatives in metropolitan São Paulo, 2016

Variables	Pain/discomfort n (%)	OR	95%CI	p-value
Cooperative				
A	39 (83)	6.66	2.78-16.67	< 0.001
B	45 (71)	3.45	1.67-7.14	
C	50 (70)	3.33	1.64-6.67	
D	29 (42)	1.00	Ref.	Ref.
Prior occupation				
No	19 (46)	1.00	Ref.	Ref.
Yes	144 (69)	2.56	1.29-5.06	0.007
Prior occupational accident				
No	93 (57)	1.00	Ref.	Ref.
Yes	70 (81)	3.34	1.78-6.23	< 0.001
Main work posture				
Seated	10 (59)	1.00	Ref.	Ref.
Standing	145 (66)	1.35	0.49-3.69	0.778
Alternating	8 (61)	1.20	0.38-3.84	0.931
Physical effort at work				
No	15 (65)	1.00	Ref.	Ref.
Yes	148 (65)	0.99	0.40-2.45	0.999
Monotony at work				
No	19 (67)	1.00	Ref.	Ref.
Yes	144 (54)	1.72	0.83-3.57	0.150
Repetitiveness at work				
No	25 (76)	1.00	Ref.	Ref.
Yes	138 (64)	1.81	0.77-4.17	0.161
Productivity count				
No	59 (62)	1.00	Ref.	Ref.
Yes	104 (67)	1.25	0.73-2.14	0.413
Shift rotation				
No	11 (65)	1.00	Ref.	Ref.
Yes	152 (67)	1.25	0.73-2.14	0.413
Rest breaks				
No	19 (59)	1.00	Ref.	Ref.
Yes	144 (66)	1.33	0.62-2.84	0.463

OR = odds ratio.

Although work organization variables (such as main work posture, physical effort, monotony, repetitiveness, productivity count, shift rotation, and rest breaks) may be risk factors for musculoskeletal pain/discomfort, none were significantly associated with it. Among the factors included in the multivariate models, only the workplace (cooperatives A, B, C or D), having a prior occupation, and having a prior occupational accident remained significant in the final models (Table 5).

**Table 5:** Association between pain/discomfort in the last 12 months and workplace, prior occupation, and prior occupational accidents among recyclable material collectors from cooperatives in metropolitan São Paulo, 2016

Variables	OR	95%CI	p-value
Cooperative			
A	5.03	2.00-12.72	0.001
B	2.93	1.39-6.21	
C	2.65	1.28-5.74	
D	1.00		
Prior occupation			
No	1.00		0.043
Yes	2.13	1.02-4.44	
Prior occupational accident			
No	1.00		0.001
Yes	2.78	1.44-5.35	

OR = odds ratio.

## DISCUSSION

This study identified a high prevalence of musculoskeletal disorders, pain, and self-reported occupational accidents among recyclable material collectors from four cooperatives in metropolitan São Paulo, Brazil. Pain has been identified as the main work-related complaint among recyclable material collectors, as a consequence of the intense work and discomfort experienced daily, which compromise their health and quality of life.^17^

The pain and occupational accident prevalence observed in other studies on Brazilian waste picker cooperatives is similar to ours,^18,19^ while other studies report a high frequency without quantifying it.^14,17,20,21^ Important factors for musculoskeletal disorders include the type of production process, the equipment conditions, and prior work history, in addition to a high workload and frequent exposure to ergonomic, mechanical, accident, physical, biological, and psychosocial risks.

Workplace visits made it clear that most cooperatives operate under precarious conditions that are unfavorable to decent and dignified work, as observed in studies of other cooperatives in Brazil.^10,22,23^ Hence, the cooperatives with worse infrastructure, working conditions, and production processes (requiring greater physical demand) had the highest proportion of musculoskeletal complaints.

Although statistical analysis revealed no significant association between self-reported pain/discomfort and most of the included risk factors (work organization, work environment, and work processes [posture, physical effort, monotony, repetitiveness, payment for productivity, rotating shifts, and pace]) they have already been identified as unfavorable conditions that can lead to accidents and musculoskeletal disorders.^10,18-20,22^ Psychosocial risk factors can also favor the occurrence of work accidents and musculoskeletal disorders. Informal employment is common among waste pickers and can lead to precarious working conditions and a greater risk of accidents and health problems.^20,22^

Some factors that contribute to informal employment among the waste pickers in this study include the way cooperatives are recruited by associations, payment mechanisms (whether through a collective or individual distribution system), work schedule, the seasonality of materials, in addition to the limited number of cooperatives and associations, the difficulties they face in continuing to operate, whether due to failure to comply with environmental requirements or to administrative and financial difficulties.^7,20,24^

In Brazil, most waste pickers work independently and without formal employment or work contracts and are responsible for supporting their families, despite the low pay. They usually do not contribute to the social security system, leaving them without little or no social protection when they need it, such as when they suffer accidents or become ill.^7,8^ However, almost all of the participants contributed to the social security system through automatic deductions from their paychecks.

Furthermore, collectors are generally dependent on processing industry regulations regarding production logistics and pricing.^7^ Another chronic problem for waste pickers is determining the amount of waste to collect and separate, which is not accounted for in their earnings, leading to unstable income and work overload.^9^ Such findings, which were also apparent in our sample, indicate the vulnerable socioeconomic conditions of these workers.

The employment relationships most commonly observed among our sample of waste collectors were association (i.e., profit sharing among workers), or individual income based on daily production or hours worked.

Although high worker turnover has been reported in cooperatives,^25-28^ we observed relative job stability in our study, similar to previous reports.^19^ Among waste collectors who reported a prior occupation, activities that also required high physical effort, such as general labor and domestic work, were the most common, as observed by Galon & Marziale.^26^

Women are a fundamental part of the recycling workforce, especially cooperatives.^14,21,22,27^ Despite the greater prevalence of female waste pickers in our sample, we observed that, in addition to the manual work of collecting and processing waste, women predominantly took on ‘internal’ and administrative functions, as did older workers. The predominance of workers who were Black or mixed race,^22,23,28^ over 30 years of age, and with low education^12,14,18,19,21^ has also been observed in other studies. These findings reflect the intense social and racial inequalities faced by recyclable material collectors.

Furthermore, a lack of labor rights and public policies contributes to the high socioeconomic vulnerability of waste collectors, as does individual vulnerability, such as low education, advanced age, gender inequality, violence, family and work conflicts, and the social stigma associated with the profession. These factors, in turn, affect worker health and contribute to the “daily occupational risks of recycling”.^17^

This study has some limitations, such as its limited and non-probabilistic sample of cooperatives linked to the National Recyclable Material Collectors Movement in metropolitan São Paulo. Nevertheless, our sample represents approximately 25% of the approximately 1,000 waste collectors working in cooperatives in the city.^5^ Memory and information bias may have affected the workers’ self-reported responses. Furthermore, the workers may have understood “occupational accidents” as serious events that caused them to miss work, since skin punctures and abrasions were rarely mentioned, although they are common, as has been pointed out in a systematic review.^22^

## CONCLUSIONS

### URGENTLY NEEDED PUBLIC INITIATIVES AND POLICIES FOR WASTE PICKERS

The challenges that governments face to support recyclable material collectors are complex and involve many spheres and actors. First, environmental education programs and strategies must be intensified, and more incisive campaigns for the correct disposal of solid waste are needed to increase the involvement of cooperatives and reduce the risk of occupational accidents and health problems.

Accident prevention must be encouraged in the work environments and work processes of waste pickers, in addition to greater social and labor protection and a transition to formal employment. Mechanisms must be established to ensure social justice and adequate remuneration for waste pickers, establishing a minimum income and recognizing their essential contribution to maintaining urban cleanliness and public health, especially in metropolitan areas. To this end, greater investment is needed in human resources and infrastructure to improve the operating conditions of cooperatives, including subsidies to purchase materials, work tools, and collective and individual protective equipment, in addition to investment in worker training, and ensuring decent and safe work environments. This should reduce the number of occupational accidents and musculoskeletal disorders and improve their health and quality of life.

As essential environments for articulating worker demands and establishing collaborations (governmental or otherwise), cooperatives and organizational networks must be supported and strengthened, especially as spaces for health education and occupational risk prevention.^24^

All health surveillance and assistance professionals, regardless of the care level, must help identify the risks of musculoskeletal disorders related to the work of waste pickers and must recommend appropriate measures for their reduction or elimination.

As gateways to the Brazilian Unified Health System, primary health care agencies, especially the *Estratégia de Saúde da Família* (Family Health Strategy) and basic health units, must welcome and recognize these workers, reinforce disease prevention and health promotion initiatives, encourage self-care and good work practices, report work-related illnesses and injuries, monitor patients, and refer them to specialized services and other health care networks when necessary. It is also important to comply with vaccination and infectious disease prevention schedules, since occupational accidents due to punctures, stings, and bites are common among waste pickers.

Occupational health surveillance agencies, such as the *Centros de Referência em Saúde do Trabalhador* (Occupational Health Reference Centers), must perform regular visits to cooperatives, including ongoing health education, training on work-related injury risks, health protection and disease prevention measures, and the use of collective and individual protective equipment, which are still little used in cooperatives. To avoid overload and reduce the risk of occupational accidents and disease, workplace engineering and administrative control measures are needed, including the reorganization of work and tasks. Waste pickers are highly vulnerable to extreme weather events and are already suffering the effects of severe climate change, such as heat waves and floods.^24^ It is also essential to advance scientific knowledge about the health risks, work environments, and work processes of recyclable material collectors, encouraging and financing further research that helps guarantee their rights and social protection.

Finally, recently implemented public policies have increased recognition for waste pickers in local selective collection and reverse logistics systems, such as being considered essential actors in the recycling chain in the National Solid Waste Policy and the inclusion of “recyclable material picker” in the Brazilian Classification of Occupations, enabling these workers to be identified in the main social, labor, and health databases. Nevertheless, effectively promoting comprehensive health, quality of life, and decent work for waste pickers is a huge challenge and is far from being achieved.
